# Causal relationship between thyroid dysfunction and hallux valgus: A two-sample Mendelian randomization study

**DOI:** 10.3389/fendo.2023.1115834

**Published:** 2023-03-08

**Authors:** Binglang Xiong, Zixing Bai, Xuhan Cao, Duorui Nie, Cheng Zhang, Xudong Sun, Ziyan Guo, Jianmin Wen, Weidong Sun

**Affiliations:** ^1^ Second Department of Orthopedics, Wangjing Hospital of China Academy of Chinese Medical Sciences, Beijing, China; ^2^ Graduate School, Hunan University of Traditional Chinese Medicine, Changsha, China; ^3^ Fourth Department of Orthopedics, Wangjing Hospital of China Academy of Chinese Medical Sciences, Beijing, China

**Keywords:** thyroid, hypothyroidism, hallux valgus, causality, Mendelian randomization analysis

## Abstract

**Introduction:**

Previous observational studies have reported that thyroid dysfunction is associated with hallux valgus (HV). However, the causal effect of thyroid dysfunction on hallux valgus is still unknown. To assess whether there is a causal relationship between thyroid dysfunction and hallux valgus, we performed a two-sample Mendelian randomization (MR) study.

**Methods:**

The data of the two-sample Mendelian randomization study were obtained from public databases. In this study, hypothyroidism, hyperthyroidism, free thyroxine (FT4), and thyrotropin (TSH) were chosen as exposures. The single nucleotide polymorphisms (SNP) of hypothyroidism and hyperthyroidism were from the genome-wide association studies (GWAS) of the IEU database, including 337,159 subjects. Data for FT4 and TSH (72,167 subjects) were extracted from the ThyroidOmics Consortium. HV was used as the outcome. The SNPs associated with HV were selected from a GWAS of 202,617 individuals in the fignngen database. The inverse variance weighted (IVW) method was used as the primary analysis. Four complementary methods were applied, including MR-presso, MR-Egger, and weighted median. In addition, Cochran’s *Q* test, MR-presso, MR-Egger regression, and the leave-one-out test were used as sensitivity analysis, and the MR-pleiotropy test was performed to examine pleiotropy.

**Results:**

According to the results of IVW, we found that there was a causal relationship between hypothyroidism and HV, and hypothyroidism increased the incidence of HV (OR = 2.838 (95% CI: 1.116–7.213); *p* = 0.028). There were no significant causal effects of hyperthyroidism, FT4, and TSH on HV (*p* > 0.05). Sensitivity analyses showed that the results were robust and reliable, and no horizontal pleiotropy was detected.

**Conclusions:**

Our findings provided genetic support that hypothyroidism might increase the risk of HV. It will predict the occurrence of HV in patients with hypothyroidism and provide suggestions for early prevention and intervention.

## Introduction

Hallux valgus (HV) is one of the most common forefoot deformities (1), which is mainly characterized by the progressive aggravation of lateral hallux deviation and medial deviation of the first metatarsal, often leading to severe foot pain and walking dysfunction (2) and thus reducing the quality of life of patients (3). According to studies, women are more likely than men to develop HV, which affects 23% of individuals between the ages of 18 and 65 and 35.7% of adults over the age of 65 (2). However, the etiology of HV is currently unclear (4). Genetic factors, improper shoe habits, inflammatory joint disease, and neuromuscular disease can all contribute to the occurrence of this disease (5, 6). There are many treatments for HV, although etiology-specific therapies are still lacking. At present, there are hundreds of surgical procedures reported in the literature to correct HV deformity, but their postoperative complication rates range from 10% to 50% (7, 8). More than 25% to 33% of patients are dissatisfied with the outcomes of surgery (9), and the high expense of surgery also adds to the burden on the medical system (10). Therefore, it is of high clinical value and economic significance to actively explore the etiology of HV and find a treatment for the etiology.

Previous large-scale observational studies have found a significant correlation between hypothyroidism and HV (11), but no research has yet confirmed whether there is a causal relationship between the two disorders. Numerous earlier investigations have demonstrated a connection between thyroid dysfunction and various orthopedic diseases. For instance, Tagoe et al. (12) revealed that patients with higher antithyroid peroxidase antibody (TPOAb) were more likely to develop chondrocalcification. Cell research confirms that abnormal thyroid hormone signaling raises the risk of osteoporosis, osteoarthritis, and other degenerative orthopedic illnesses (13, 14). Other studies have demonstrated that thyroid hormones can affect the function of osteoblasts and osteoclasts (15). However, hallux valgus is a common orthopedic disease, and whether thyroid disease affects it has not been explored.

Based on previous studies, we hypothesize that there may be a causal relationship between thyroid dysfunction and the risk of HV, and in this study, two-sample Mendelian randomization (MR) analysis was used to verify this. MR is an epidemiological statistical method that uses genetic variation as an instrumental variable (IV) to infer causal relationships between exposures and outcomes (16). Because genetic variation follows Mendel’s second law and is randomly assigned when fertilized eggs are formed, MR can reduce the interference of confounding factors on the results compared with previous studies and achieve the same effect as randomized controlled trials (17). MR also overcomes reverse causation because genetic variation is not affected by disease status (18). In this study, we considered hypothyroidism, hyperthyroidism, free thyroxine (FT4), and thyrotropin (TSH) as exposure and HV as outcome. This is the first study on the causal relationship between thyroid dysfunction and HV, with the purpose of further studying the etiology of HV and providing new ideas for the clinical treatment of HV.

## Materials and methods

### Study design and data sources

**Figure 1:**
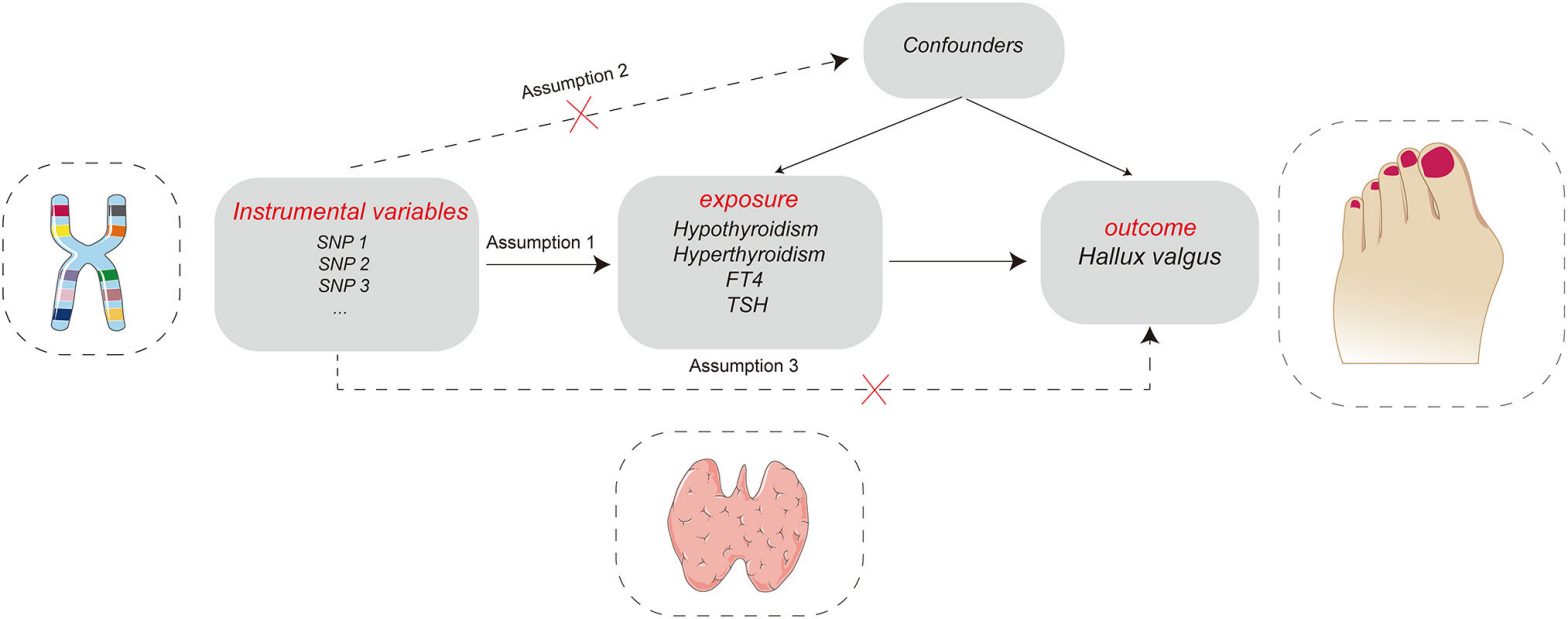
Key assumptions of the Mendelian randomization study: Assumption 1: instrumental variables should be robustly associated with exposure. Assumption 2: instrumental variables should not be associated with any confounders. Assumption 3: instrumental variables must not be associated with hallux valgus except through exposure.

We aimed to investigate the causal relationship between thyroid dysfunction and the risk of HV. Because all of the data in this study were obtained from public databases, no consent was required from the participants. We reported our study according to the STROBE-MR statement (19). The key assumptions of the MR study can be seen in **Figure 1**.

In this study, hypothyroidism (increased TSH), hyperthyroidism (decreased TSH), FT4, and TSH were chosen as exposures. The single nucleotide polymorphisms (SNP) of hypothyroidism and hyperthyroidism were from the genome-wide association studies (GWAS) of the IEU database (https://gwas.mrcieu.ac.uk/), including 337,159 subjects and 10,894,596 SNPs. The study included 16,376 hypothyroidism samples and 320,783 control samples. There were also 2,547 hyperthyroidism cases and 334,612 ncases. The GWAS data of FT4 and TSH are all from The ThyroidOmics Consortium database (https://transfer.sysepi.medizin.uni-greifswald.de/thyroidomics/datasets/), containing 72,167 samples (**Table 1**).

**Table 1:** Sources of GWAS data for instrumental variables.

Exposure	Outcome	Consortium	Population	Sample size
Hypothyroidism	HV	IEU	European	337,159
Hyperthyroidism	HV	IEU	European	337,159
FT4	HV	The ThyroidOmics Consortium	European	72,167
TSH	HV	The ThyroidOmics Consortium	European	72,167

FT4, free thyroxine; TSH, thyrotropin; HV, hallux valgus.

HV was selected as the outcome, and its GWAS data were obtained from the FinnGen database (https://www.finngen.fi/en), which included 20,2617 samples (12,055 cases, 190,562 ncases) and 16,383,115 SNPs. All participants were of European ancestry.

### Genetic IV selection

**Figure 2:**
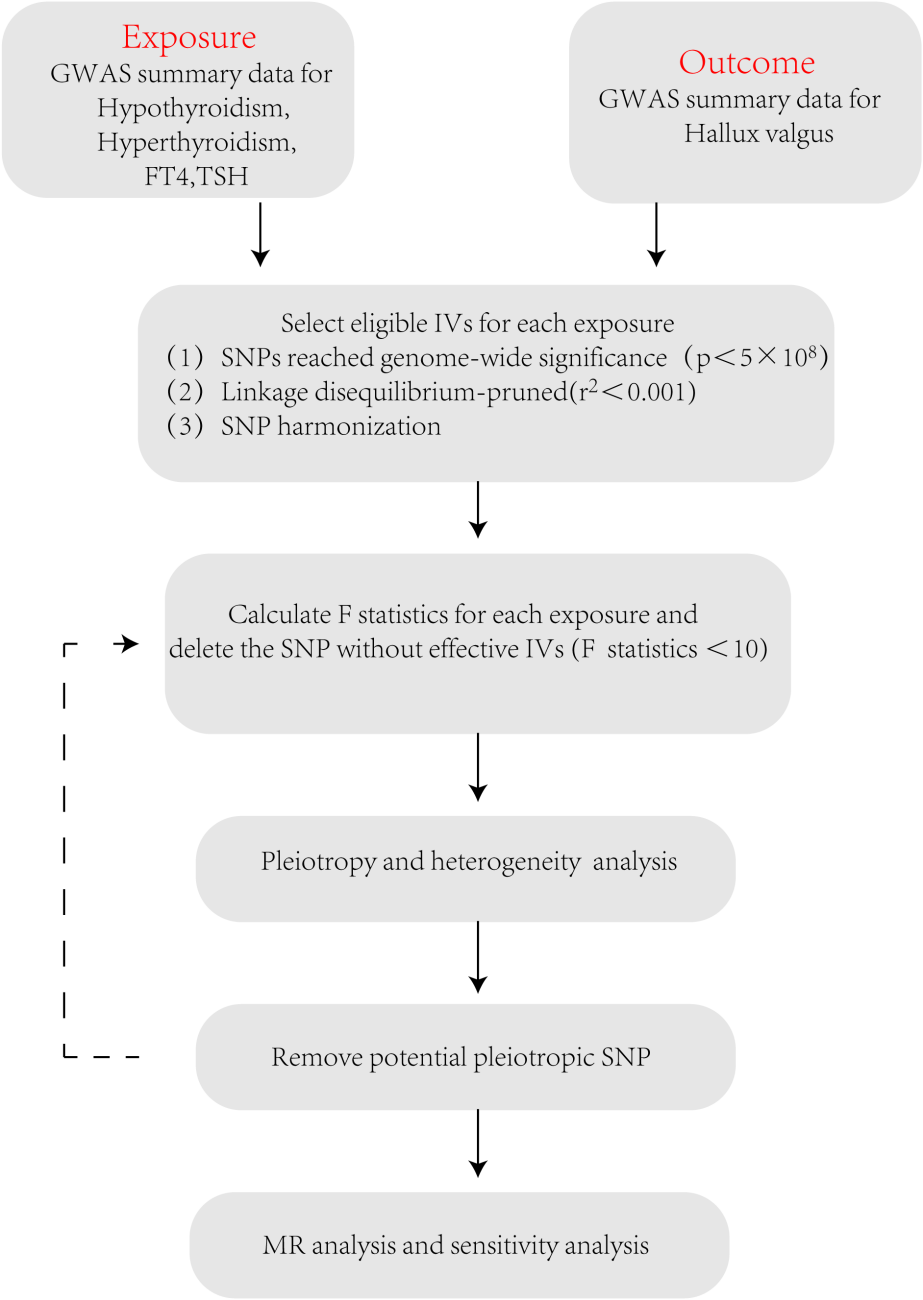
Flowchart of MR analysis in this study.

We selected effective instrumental variables (IV) based on three assumptions (**Figure 1**). First, we set that each IV was significantly correlated with exposure (*p*< 5 × 10^−8^ means that the instrumental variable is strongly correlated with exposure). To remove the linkage disequilibrium between each SNP, we set the distance to 10,000 KB and the LD *r* ^2^ to< 0.001 (20). To remove the possible horizontal pleiotropy of IV, use the Phenoscanner (http://www.phenoscanner.medschl.cam.ac.uk/) to search for phenotypes that may be affected by each SNP and remove the SNPs related to HV-associated phenotypes (21). SNPs for exposure and outcome should be harmonised, and palindromic and incompatible alleles should be removed (22). Finally, calculate the *F* value of each SNP ( $F=\beta_{exposure}^{2}/SE_{exposure}^{2}$ ), and the SNPs with an *F* value<10 should be removed (23) (**Figure 2**).

### Statistical analyses

All two-sample MR data analyses in this study were based on the TwoSampleMR package in the R software (version 4.5.0).

We used inverse variance weighting (IVW) as the primary method to evaluate the causal relationship between thyroid dysfunction and the risk of HV (24). IVW assumes that all selected IV are valid, so it has the highest statistical power and can provide the most accurate results (18). In addition, MR-Egger (25) and weighted median (26) were selected as supplementary methods. If all included SNPs match the effective IV assumption, then IVW can be considered the most reliable result (27). All results are expressed as OR values and 95% confidence intervals, with *p*< 0.05 representing statistical significance.

### Sensitivity analysis

Cochrane’s *Q* was used as a heterogeneity test; *p*< 0.05 represents the existence of heterogeneity (28). MR-Egger regression was used to detect horizontal pleiotropy. The intercept value of MR-Egger regression represents the strength of horizontal pleiotropy, and a *p*-value of > 0.05 means that there is no horizontal pleiotropy (29). MR-PRESSO was used to detect SNPs that may lead to pleiotropic effects, remove outlier SNPs, and then perform MR analysis to compare whether the results have changed before and after correction (30). The leave-one-out test was used to detect the robustness of the results. This method gradually eliminated a single SNP and performed MR analysis on the remaining SNPs to detect whether the single SNP had a significant impact on the results.

## Results

After a series of quality evaluations, the number of SNPs selected as effective IV in hypothyroidism, hyperthyroidism, FT4, and TSH was 70, 4, 14, and 36, respectively. In the **Supplementary Material**, information on SNPs as IV was provided (**Supplementary Tables S1–S4**). The *F* values of all SNPs used as IV are greater than 10 (**Supplementary Tables S1–S4**), indicating that the included IV perform effectively.

### Two-sample MR analysis for evaluating causal effects of FT4, TSH, hyperthyroidism, and hypothyroidism on HV

**Figure 3:**
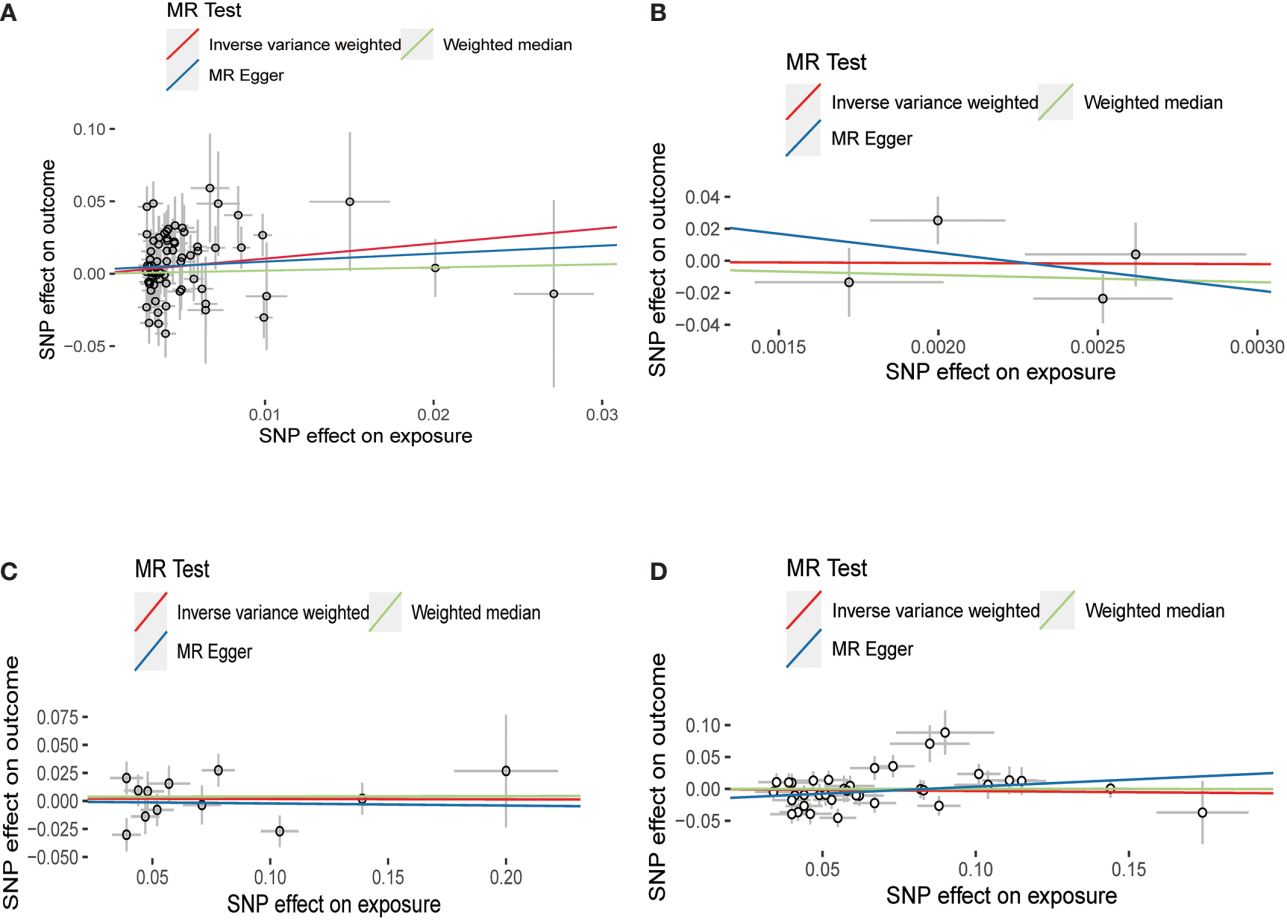
Scatter plots for Mendelian randomization (MR) analyses of the causal relationship between thyroid dysfunction and hallux valgus. **(A)** Hypothyroidism-HV. **(B)** Hyperthyroidism-HV. **(C)** FT4-HV. **(D)** TSH-HV.

IVW, MR-Egger, and weighted median methods were used to assess whether there is a causal relationship between hypothyroidism, hyperthyroidism, FT4, TSH, and the risk of HV. According to IVW results, we found that there is a positive causal relationship between hypothyroidism and HV; hypothyroidism can increase the risk of HV (OR = 2.838, 95% CI: 1.116–7.213); *p* = 0.028) (**Table 2**; **Figure 3**), and there is no statistical difference in MR-Egger and weighted median (**Table 2**; **Figure 3**). At the same time, these three methods showed no statistical significance in the assessment of the causal relationship between hyperthyroidism, FT4, TSH, and the risk of HV (**Table 2** **;** **Figure 3**).

**Table 2:** MR estimates from different methods of assessing the causal effect of thyroid dysfunction on HV.

Exposure	MR methods	nSNP	Beta	OR (95% cl)	*p*-value
Hypothyroidism	IVW	70	1.043	2.838 (1.116, 7.213)	0.028
	MR-Egger	70	0.549	1.732 (0.230, 13.004)	0.594
	Weighted median	70	0.215	1.241 (0.369, 4.169)	0.727
Hyperthyroidism	IVW	4	−0.701	0.496 (1.733e−05, 1.419e+4)	0.893
	MR-Egger	4	−23.574	5.774e−11 (1.014e−44, 3.286e+23)	0.612
	Weighted median	4	−4.459	0.012 (7.848e−07, 1.704e+2)	0.361
FT4	IVW	12	−0.003	0.996 (0.863, 1.149)	0.961
	MR-Egger	12	−0.018	0.981 (0.700, 1.376)	0.917
	Weighted median	12	0.003	1.003 (0.847, 1.188)	0.969
TSH	IVW	36	−0.035	0.965 (0.862,1.079)	0.535
	MR-Egger	36	0.219	1.246 (0.949,1.634)	0.121
	Weighted median	36	−0.002	0.998 (0.876,1.138)	0.982

FT4, free thyroxine; TSH, thyrotropin; HV, Hallux valgus; SNP, single nucleotide polymorphism; MR, Mendelian randomization; IVW, inverse variance weighting.

### Heterogeneity, pleiotropy, and sensitivity analysis

**Figure 4:**
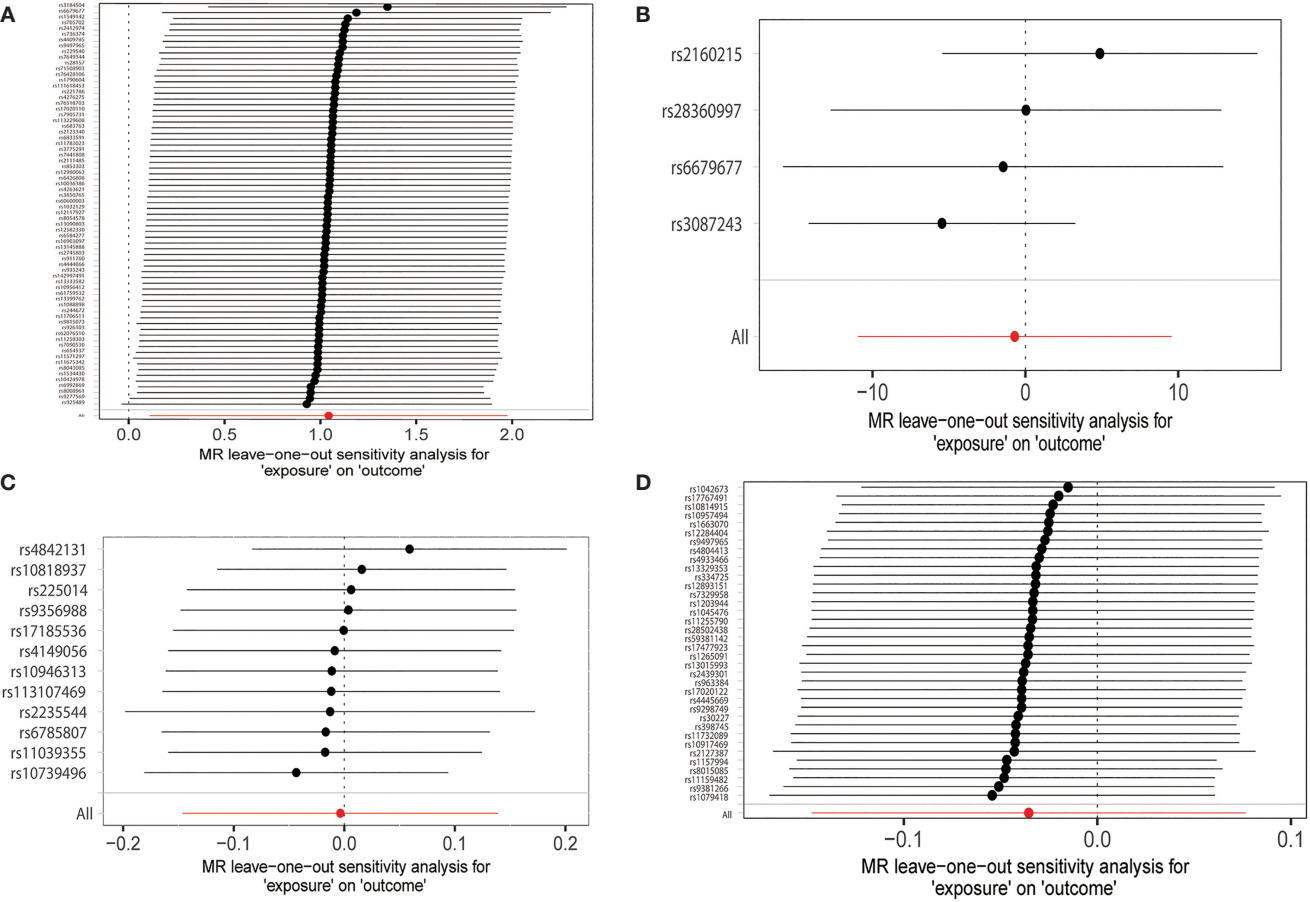
MR leave-one-out sensitivity analyses of the causal relationship between thyroid dysfunction and hallux valgus. **(A)** Hypothyroidism-HV. **(B)** Hyperthyroidism-HV. **(C)** FT4-HV. **(D)** TSH-HV.

All the results of this part are presented in **Table 3**. We found no heterogeneity when hyperthyroidism (*p* = 0.131) and FT4 (*p* = 0.132) were used as exposure. However, when hypothyroidism (*p* = 0.001) and TSH (*p* = 0.0004) were considered exposures, we discovered heterogeneity. The sources of these heterogeneities may be due to differences in the source of SNP data, experimental conditions, detection methods, and included populations. When heterogeneity was present, the random-effects model in IVW was chosen (30). MR-Egger regression showed that there was no horizontal pleiotropy for the SNPs of all exposures (**Table 3**). Neither MR-presso found outliers, and the results were consistent with IVW results (**Table 3**). the leave-one-out test further confirms that the results are stable, as shown in **Figure 4**. Funnel plots can be seen in **Supplementary Figure S1**, and the forest plots in MR analysis are shown in **Supplementary Figure S2**. Therefore, we considered the results of IVW to be reliable.

**Table 3:** Sensitivity analysis of thyroid dysfunction causally linked to HV.

Exposure	Outcome	Pleiotropy		Heterogeneity		Outlier examination by MR-PRESSO	
		Horizontal pleiotropy (Egger intercept)	Horizontal pleiotropy (*p*-value)	Heterogeneity (*Q*)	Heterogeneity (*p*-value)	Before correction (*p*-value)	After correction (*p*-value)
Hypothyroidism	HV	0.002	0.589	109.245	0.001	0.032	NA
Hyperthyroidism	HV	0.052	0.618	5.648	0.130	0.902	NA
FT4	HV	0.001	0.925	16.229	0.132	0.962	NA
TSH	HV	−0.018	0.052	69.531	0.0004	0.539	NA

FT4, free thyroxine; TSH, thyrotropin; HV, hallux valgus; MR, Mendelian randomization.

## Discussion

In our two-sample MR study, we found a positive causal relationship between hypothyroidism and HV, with hypothyroidism associated with an increased risk of HV. However, there is no obvious causal relationship between hyperthyroidism, FT4, TSH, and HV. Our findings support the results of a previous observational study. Sterling et al. (11) investigated the prevalence of thyroid disease in 350 patients with forefoot deformity who visited the doctor for the first time and found that the prevalence of hypothyroidism was the highest among patients (19.1%) and that patients with hallux valgus were more likely to have thyroid disease (61.5%). Subsequently, the researchers analyzed a national public database of 905,924 patients with forefoot deformities and found that 321,656 (35.5%) patients were diagnosed with thyroid disease. Finally, it was concluded that forefoot deformities, especially HV, were significantly associated with thyroid dysfunction (11). A limitation of this study is that it did not examine the causal relationship between thyroid dysfunction and hallux valgus, which our findings provide support for. Our findings provide a new perspective on the etiology of hallux valgus, which is that hypothyroidism may contribute to the development of HV.

A large number of experimental studies have confirmed that bones are sensitive to thyroid hormones, and the biological role of thyroid hormones in bone tissue and cartilage has also attracted more and more attention (13). However, the specific biological mechanism behind the positive causal relationship between hypothyroidism and HV remains unclear. Previous literature holds that the thyroid hormone is an important regulator of bone metabolism and is crucial for bone remodeling; any deficiency or excess may lead to bone metabolism disorder (31). Hyperthyroidism and TSH levels lower than normal can accelerate bone metabolism and lead to accelerated bone loss (32, 33). In contrast, histomorphological data from hypothyroid patients showed decreased bone turnover and increased bone calcification (34). The study by Robinson et al. (35) found that there may be a specific cell population in the soft tissue surrounding HV, and cell experiments proved that when stimulated by fibroblast growth factor (FGF), it would lead to increased osteogenesis and the formation of HV. From this, we can speculate that patients with hypothyroidism may suffer from HV due to regionally impaired bone metabolism. Sclerostin, an osteocyte-derived protein encoded by the SOST gene, acts as an inhibitor of bone formation by stimulating the apoptosis of osteoblasts (36). Studies have shown that the content of sclerostin in the blood circulation of patients with hyperthyroidism is significantly higher than that of patients with hypothyroidism, and the level of sclerostin is positively correlated with FT4 and negatively correlated with TSH (37). This provides further evidence that hypothyroid patients are more likely to exhibit osteogenic dominance.

Some studies have studied the distribution of thyroid hormone receptors in human bone tissue. They found that at sites of endochondral ossification in osteophytes, TRalpha1, TRalpha2 variants, TRbeta1, and TRbeta2 mRNA were widely distributed in undifferentiated, proliferating, mature, and hypertrophic chondrocytes. Most osteoblasts (> 90%) express TRalpha-1 mRNA at regions of bone remodeling (38). A part of the concurrent clinical symptoms of HV is the formation of osteophytes, and studies have shown that there is a significant correlation between the cartilage degeneration of the first metatarsophalangeal joint and the severity of hallux valgus (39). Therefore, we can speculate that hypothyroidism may mediate cartilage degeneration leading to hallux valgus.

Other studies have found that the synovium of patients with rheumatoid arthritis (RA) and osteoarthritis (OA) contains a network of thyroid hormones. Thyroid hormones are strongly biodegraded in the synovium, and synoviocytes play an important role in the activation and degradation of thyroid hormones (40). HV is frequently present alongside synovitis, which is strongly connected with pain in this condition (41, 42). This leads us to hypothesize that a series of pathological changes caused by the action of thyroid hormone on the synovium may also contribute to the development of hallux valgus. However, the above speculations on the mechanism are all based on existing research results. No studies have investigated the mechanism of thyroid hormones on HV, which may be the subject of our upcoming study.

Most of the previous knowledge about the etiology of HV is that abnormalities in anatomy or biomechanics lead to the occurrence of the disease (43). No studies have investigated endocrine disorders as an etiology of HV. The surgical approaches for treating HV are varied since there are numerous pathologic variables, and the choice of surgical method is often based on the preference of the surgeon (5). However, the treatment satisfaction of the operation is poor (9). No consensus has been reached about the gold standard of HV treatment, despite the fact that there are several studies comparing the effectiveness of various surgical techniques (44, 45). However, there are still a large number of patients waiting for treatment every year. A 1994 study estimated that there are approximately 209,000 HV operations in the USA each year (46). Therefore, it is particularly important to actively explore other possible causes and establish a standardized treatment system. Our research suggests that hypothyroidism may be one of the causes of HV, and people with hypothyroidism are 2.838 times more likely to develop HV than normal people. Based on the results of this study, it may provide new ideas for the treatment of HV and provide a theoretical basis for the development of drug therapy for HV in the future. Early treatment of hypothyroid patients may reduce the incidence of hallux valgus and improve the quality of life of patients. Thyroid function screening and timely intervention in the HV patient population may slow the progression of HV and avoid the eventual development of surgery. In the USA, per HV surgery costs about $18,332 (10). Numerous surgical expenditures can be avoided if the amount of operations is decreased. Therefore, our findings have important implications for public health.

There are some advantages to our study. First of all, compared with observational studies, we use MR studies for causal inference and select genetic variation as IV, which can avoid the interference of other confounding factors and reverse causality. Second, all samples were drawn from European populations, and our results are less susceptible to differences in demographics. Most importantly, we demonstrate for the first time a causal relationship between hypothyroidism and HV, which may provide new strategies for the treatment of HV.

There are also some deficiencies in this study. First, thyroid hormones include multiple subtypes, such as T3 and T4, but due to research limitations, we cannot obtain the SNP data of the remaining subtypes. We were not able to explore other thyroid hormone subtypes as exposures for causal effects on HV. Second, FT4 and TSH are often used as indicators for the diagnosis of hypothyroidism, but they have not shown a causal relationship with hallux valgus. This may be caused by a relatively insufficient sample size of FT4 and TSH. Third, the participants we included were all from European populations, so it is not clear whether our findings are applicable to other populations. In addition, some of the methods we used in this study did not produce the same results as IVW, but all of the SNPs included in our study met the assumption of effective IV, and the MR-presso results were consistent with IVW, so our results are still reliable.

## Conclusion

We used the two-sample MR to study the causal relationship between thyroid function and HV and proved that there is a positive causal relationship between hypothyroidism and HV. Hypothyroidism could increase the risk of HV. It will predict the occurrence of HV in patients with hypothyroidism and provide suggestions for early prevention and intervention. However, the results of this study need to be further confirmed by basic experiments.

## Data availability statement

The original contributions presented in the study are included in the article/**Supplementary Material**. Further inquiries can be directed to the corresponding author.

## Ethics statement

Ethical review and approval was not required for the study on human participants in accordance with the local legislation and institutional requirements. Written informed consent for participation was not required for this study in accordance with the national legislation and the institutional requirements.

## Author contributions

BX: research design and paper writing. ZB, XC, and DN: collection and organization of data. CZ, XS, and ZG: analysis and visualization of results. JW and WS: research quality assessment. All authors contributed to the article and approved the submitted version.
