# Supplementary material for: Causal relationship between thyroid dysfunction and hallux valgus: A two-sample Mendelian randomization study
Source: Front Endocrinol (Lausanne). 2023 Mar 8;14:1115834. doi: 10.3389/fendo.2023.1115834 (PMC10030973; doi:10.3389/fendo.2023.1115834)
Supplement: Supplementary file 1 [file DataSheet_1.docx]

**TABLE S1 Univariate Mendelian randomization analysis for the effects of Hypothyroidism on venous Hallux valgus risk.**

| **SNP** | **EAE** | **OAE** | **EAO** | **OAO** | **exposure** | | | **outcome** | | | **F** |
| --- | --- | --- | --- | --- | --- | --- | --- | --- | --- | --- | --- |
|  |  |  |  |  | **se** | **beta** | **pval** | **se** | **beta** | **pval** |  |
| rs10036386 | T | C | T | C | 0.000534 | 0.003214 | 1.75E-09 | 0.014159 | 0.001677 | 0.905695 | 36 |
| rs1032129 | C | A | C | A | 0.000544 | -0.00302 | 2.67E-08 | 0.014264 | -0.00567 | 0.690734 | 31 |
| rs10424978 | A | C | A | C | 0.000535 | -0.00416 | 7.19E-15 | 0.014311 | -0.02935 | 0.040276 | 61 |
| rs1088898 | T | G | T | G | 0.000618 | 0.003386 | 4.22E-08 | 0.015311 | 0.022771 | 0.136942 | 30 |
| rs10956412 | C | A | C | A | 0.000706 | -0.00509 | 5.83E-13 | 0.023965 | -0.03169 | 0.186099 | 52 |
| rs111618453 | A | G | A | G | 0.000583 | 0.005774 | 4.30E-23 | 0.015353 | -0.00362 | 0.813824 | 98 |
| rs11258303 | A | C | A | C | 0.000599 | 0.003713 | 5.58E-10 | 0.014724 | 0.025005 | 0.089466 | 38 |
| rs113229608 | A | C | A | C | 0.001064 | 0.006475 | 1.17E-09 | 0.0369 | -0.02513 | 0.495842 | 37 |
| rs11571297 | C | T | C | T | 0.000521 | -0.00859 | 5.05E-61 | 0.01445 | -0.01805 | 0.21154 | 272 |
| rs11675342 | T | C | T | C | 0.000525 | 0.004611 | 1.66E-18 | 0.014149 | 0.022119 | 0.117998 | 77 |
| rs11706511 | G | A | G | A | 0.000781 | 0.004659 | 2.47E-09 | 0.019938 | 0.033317 | 0.094709 | 36 |
| rs11783023 | T | C | T | C | 0.000576 | -0.0033 | 1.06E-08 | 0.014518 | 0.002385 | 0.869498 | 33 |
| rs12117927 | A | C | A | C | 0.000532 | 0.003269 | 8.17E-10 | 0.014525 | 0.005851 | 0.687086 | 38 |
| rs12582330 | T | G | T | G | 0.000585 | -0.00416 | 1.10E-12 | 0.015004 | -0.00855 | 0.568818 | 51 |
| rs12980063 | G | A | G | A | 0.000532 | -0.0034 | 1.73E-10 | 0.014644 | -0.00059 | 0.967666 | 41 |
| rs13090803 | T | G | T | G | 0.000639 | 0.005071 | 2.09E-15 | 0.020258 | 0.011131 | 0.582697 | 63 |
| rs13145888 | C | T | C | T | 0.000634 | -0.00601 | 2.44E-21 | 0.021519 | -0.01575 | 0.464182 | 90 |
| rs13333582 | C | T | C | T | 0.001282 | 0.007221 | 1.80E-08 | 0.036086 | 0.048426 | 0.179604 | 32 |
| rs13399762 | G | A | G | A | 0.001145 | 0.006728 | 4.24E-09 | 0.037481 | 0.05915 | 0.114535 | 35 |
| rs142997491 | G | A | G | A | 0.002382 | 0.01503 | 2.81E-10 | 0.047889 | 0.049732 | 0.299047 | 40 |
| rs1534430 | T | C | T | C | 0.000533 | -0.00403 | 3.98E-14 | 0.014184 | -0.02776 | 0.050349 | 57 |
| rs1549142 | T | C | T | C | 0.000619 | 0.004091 | 3.89E-11 | 0.016445 | -0.04136 | 0.011909 | 44 |
| rs16903097 | G | T | G | T | 0.000769 | -0.00452 | 3.97E-09 | 0.02188 | -0.01612 | 0.461362 | 35 |
| rs17020110 | C | T | C | T | 0.000588 | 0.00413 | 2.06E-12 | 0.016705 | -0.00666 | 0.690316 | 49 |
| rs1790604 | G | A | G | A | 0.00052 | -0.00323 | 5.48E-10 | 0.014106 | 0.011577 | 0.411836 | 38 |
| rs2111485 | G | A | G | A | 0.000531 | 0.003802 | 7.83E-13 | 0.014164 | 0.000538 | 0.96969 | 51 |
| rs2123340 | A | G | A | G | 0.000546 | -0.00374 | 7.75E-12 | 0.014753 | 0.003701 | 0.801902 | 47 |
| rs221786 | C | T | C | T | 0.000819 | 0.00495 | 1.53E-09 | 0.019576 | -0.01226 | 0.531057 | 36 |
| rs229540 | G | T | G | T | 0.000525 | 0.005037 | 8.77E-22 | 0.014245 | -0.01088 | 0.445025 | 92 |
| rs2412974 | T | C | T | C | 0.000541 | -0.00312 | 8.00E-09 | 0.014254 | 0.033962 | 0.017187 | 33 |
| rs244672 | T | C | T | C | 0.000791 | -0.00463 | 4.84E-09 | 0.016389 | -0.02097 | 0.20071 | 34 |
| rs2745803 | G | A | G | A | 0.000641 | -0.00368 | 9.31E-09 | 0.019705 | -0.02024 | 0.304416 | 33 |
| rs28157 | T | G | T | G | 0.000559 | -0.00349 | 4.16E-10 | 0.015041 | 0.018979 | 0.207 | 39 |
| rs3184504 | C | T | C | T | 0.000519 | -0.00993 | 9.99E-82 | 0.014224 | 0.030264 | 0.033364 | 367 |
| rs3775291 | T | C | T | C | 0.000566 | -0.00403 | 1.05E-12 | 0.015134 | 0.000789 | 0.958427 | 51 |
| rs3850765 | C | T | C | T | 0.000526 | 0.00313 | 2.76E-09 | 0.015399 | 0.00503 | 0.743942 | 35 |
| rs4263621 | A | G | A | G | 0.000522 | 0.002956 | 1.47E-08 | 0.014047 | 0.001375 | 0.922036 | 32 |
| rs4276275 | T | C | T | C | 0.00052 | 0.003404 | 5.83E-11 | 0.013994 | -0.00771 | 0.581455 | 43 |
| rs4409785 | C | T | C | T | 0.000687 | 0.00648 | 4.28E-21 | 0.018801 | -0.02081 | 0.268258 | 89 |
| rs4444866 | T | C | T | C | 0.000582 | -0.00323 | 2.85E-08 | 0.015483 | -0.01545 | 0.31848 | 31 |
| rs60600003 | G | T | G | T | 0.000865 | 0.005003 | 7.19E-09 | 0.023209 | 0.008356 | 0.71882 | 33 |
| rs61759532 | T | C | T | C | 0.000619 | 0.004192 | 1.26E-11 | 0.017955 | 0.022749 | 0.205147 | 46 |
| rs62076510 | G | T | G | T | 0.000715 | 0.0052 | 3.45E-13 | 0.018707 | 0.028777 | 0.12397 | 53 |
| rs6426808 | A | G | A | G | 0.00052 | 0.003272 | 3.12E-10 | 0.014022 | 0.000712 | 0.959477 | 40 |
| rs654537 | A | G | A | G | 0.000532 | 0.005993 | 2.11E-29 | 0.013977 | 0.018656 | 0.181947 | 127 |
| rs6584277 | G | A | G | A | 0.000519 | -0.00318 | 8.79E-10 | 0.014001 | -0.00985 | 0.481819 | 38 |
| rs6679677 | A | C | A | C | 0.000853 | 0.020102 | 8.47E-123 | 0.019872 | 0.003985 | 0.841047 | 555 |
| rs6833591 | G | A | G | A | 0.000546 | -0.00317 | 6.09E-09 | 0.017 | 0.005604 | 0.741667 | 34 |
| rs683763 | T | G | T | G | 0.000555 | 0.003091 | 2.57E-08 | 0.014296 | -0.00568 | 0.690965 | 31 |
| rs6992869 | C | T | C | T | 0.000537 | 0.002991 | 2.56E-08 | 0.014107 | 0.046223 | 0.001051 | 31 |
| rs705702 | G | A | G | A | 0.000548 | 0.00368 | 1.96E-11 | 0.015261 | -0.0345 | 0.0238 | 45 |
| rs7090530 | A | C | A | C | 0.00053 | 0.004187 | 2.93E-15 | 0.01493 | 0.024401 | 0.102195 | 62 |
| rs71508903 | T | C | T | C | 0.000662 | 0.006257 | 3.19E-21 | 0.018122 | -0.01043 | 0.565045 | 89 |
| rs736374 | A | G | A | G | 0.000541 | 0.004121 | 2.47E-14 | 0.014685 | -0.02242 | 0.126905 | 58 |
| rs7441808 | G | A | G | A | 0.000564 | 0.003541 | 3.53E-10 | 0.01552 | -0.00114 | 0.941203 | 39 |
| rs76428106 | C | T | C | T | 0.00238 | 0.027135 | 4.20E-30 | 0.064492 | -0.01389 | 0.829514 | 130 |
| rs7649344 | C | T | C | T | 0.000521 | -0.00297 | 1.23E-08 | 0.014476 | 0.023166 | 0.109528 | 32 |
| rs76518703 | G | A | G | A | 0.001221 | -0.01009 | 1.42E-16 | 0.037133 | 0.015526 | 0.675868 | 68 |
| rs7905731 | C | T | C | T | 0.000528 | -0.00314 | 2.66E-09 | 0.014585 | 0.007094 | 0.626679 | 35 |
| rs8008961 | T | C | T | C | 0.000578 | -0.00337 | 5.53E-09 | 0.01525 | -0.04852 | 0.001466 | 34 |
| rs8043085 | T | G | T | G | 0.000614 | 0.004251 | 4.46E-12 | 0.016659 | 0.030915 | 0.063486 | 48 |
| rs8054578 | G | A | G | A | 0.000623 | -0.00358 | 8.94E-09 | 0.018484 | -0.00852 | 0.645008 | 33 |
| rs853303 | G | A | G | A | 0.000534 | -0.00375 | 2.30E-12 | 0.015165 | -0.00058 | 0.969325 | 49 |
| rs911760 | A | C | A | C | 0.000673 | 0.004181 | 5.09E-10 | 0.017211 | 0.015848 | 0.357156 | 39 |
| rs925489 | T | C | T | C | 0.000551 | 0.009857 | 1.41E-71 | 0.014706 | 0.026651 | 0.069958 | 320 |
| rs926103 | C | T | C | T | 0.000548 | -0.00299 | 4.92E-08 | 0.014433 | -0.02724 | 0.05913 | 30 |
| rs9277569 | T | C | T | C | 0.00084 | 0.008408 | 1.35E-23 | 0.020006 | 0.040468 | 0.043088 | 100 |
| rs933243 | A | C | A | C | 0.00055 | -0.00557 | 3.86E-24 | 0.014476 | -0.01263 | 0.382901 | 103 |
| rs9497965 | T | C | T | C | 0.000529 | 0.003647 | 5.18E-12 | 0.014894 | -0.02686 | 0.071359 | 48 |
| rs9815073 | A | C | A | C | 0.000568 | -0.00706 | 1.90E-35 | 0.015004 | -0.01789 | 0.233175 | 154 |

SNP: single nucleotide polymorphism; EAE: effect allele exposure; OAE: other allele exposure; EAO: effect allele outcome; OAO: other allele outcome; SE: standard error;F:F value;

**TABLE S2 Univariate Mendelian randomization analysis for the effects of Hyperthyroidism on venous Hallux valgus risk.**

| **SNP** | **EAE** | **OAE** | **EAO** | **OAO** | **exposure** | | | **outcome** | | | **F** |
| --- | --- | --- | --- | --- | --- | --- | --- | --- | --- | --- | --- |
|  |  |  |  |  | **se** | **beta** | **pval** | **se** | **beta** | **pval** |  |
| rs2160215 | C | T | C | T | 0.000218 | 0.002516 | 8.90E-31 | 0.015366 | -0.02367 | 0.123412 | 133 |
| rs28360997 | A | G | A | G | 0.000296 | -0.00172 | 6.01E-09 | 0.021445 | 0.013463 | 0.530161 | 34 |
| rs3087243 | A | G | A | G | 0.000212 | -0.002 | 3.80E-21 | 0.014861 | -0.02519 | 0.090016 | 89 |
| rs6679677 | A | C | A | C | 0.000347 | 0.002618 | 4.42E-14 | 0.019872 | 0.003985 | 0.841047 | 57 |

SNP: single nucleotide polymorphism; EAE: effect allele exposure; OAE: other allele exposure; EAO: effect allele outcome; OAO: other allele outcome; SE: standard error;F:F value;

**TABLE S3 Univariate Mendelian randomization analysis for the effects of FT4 on venous Hallux valgus risk.**

| SNP | EAE | OAE | EAO | OAO | exposure | | | outcome | | | F |
| --- | --- | --- | --- | --- | --- | --- | --- | --- | --- | --- | --- |
|  |  |  |  |  | se | beta | pval | se | beta | pval |  |
| rs10739496 | T | C | T | C | 0.007 | 0.078 | 7.76E-29 | 0.014695 | 0.027351 | 0.062706 | 124 |
| rs10818937 | C | T | C | T | 0.006 | 0.039 | 8.03E-11 | 0.014925 | -0.03017 | 0.043235 | 42 |
| rs10946313 | T | C | T | C | 0.006 | 0.044 | 2.24E-13 | 0.01413 | 0.009338 | 0.508703 | 54 |
| rs11039355 | C | T | C | T | 0.007 | 0.039 | 2.53E-08 | 0.014652 | 0.02046 | 0.162596 | 31 |
| rs113107469 | T | C | T | C | 0.022 | 0.2 | 9.82E-20 | 0.050309 | 0.026774 | 0.594595 | 83 |
| rs17185536 | T | C | T | C | 0.008 | 0.071 | 6.99E-19 | 0.017439 | -0.00358 | 0.837436 | 79 |
| rs2235544 | A | C | A | C | 0.007 | 0.139 | 9.56E-88 | 0.014103 | 0.001912 | 0.892172 | 394 |
| rs225014 | T | C | T | C | 0.006 | 0.047 | 4.75E-15 | 0.015686 | -0.01382 | 0.378403 | 61 |
| rs4149056 | C | T | C | T | 0.007 | 0.048 | 7.03E-12 | 0.017461 | 0.008618 | 0.62164 | 47 |
| rs4842131 | C | T | C | T | 0.008 | 0.104 | 1.22E-38 | 0.014065 | -0.027 | 0.054936 | 169 |
| rs6785807 | G | A | G | A | 0.009 | 0.057 | 2.40E-10 | 0.015914 | 0.015468 | 0.331072 | 40 |
| rs9356988 | G | A | G | A | 0.007 | 0.052 | 1.10E-13 | 0.014459 | -0.00796 | 0.582068 | 55 |

SNP: single nucleotide polymorphism; EAE: effect allele exposure; OAE: other allele exposure; EAO: effect allele outcome; OAO: other allele outcome; SE: standard error;F:F value;

**TABLE S4 Univariate Mendelian randomization analysis for the effects of TSH on venous Hallux valgus risk.**

| **SNP** | **EAE** | **OAE** | **EAO** | **OAO** | **exposure** | | | **outcome** | | | **F** |
| --- | --- | --- | --- | --- | --- | --- | --- | --- | --- | --- | --- |
|  |  |  |  |  | **se** | **beta** | **pval** | **se** | **beta** | **pval** |  |
| rs1042673 | G | A | G | A | 0.006 | 0.055 | 4.88E-20 | 0.014134 | -0.04566 | 0.001236 | 84 |
| rs1045476 | A | G | A | G | 0.008 | 0.049 | 9.07E-10 | 0.017919 | -0.0098 | 0.584485 | 38 |
| rs1079418 | A | G | A | G | 0.007 | 0.101 | 3.42E-47 | 0.015907 | 0.023276 | 0.143402 | 208 |
| rs10814915 | T | C | T | C | 0.006 | 0.042 | 2.56E-12 | 0.014004 | -0.03667 | 0.008826 | 49 |
| rs10917469 | A | G | A | G | 0.009 | 0.111 | 5.99E-35 | 0.021566 | 0.013223 | 0.539773 | 152 |
| rs10957494 | G | A | G | A | 0.007 | 0.04 | 1.10E-08 | 0.015199 | -0.03971 | 0.008979 | 33 |
| rs11159482 | T | C | T | C | 0.013 | 0.085 | 6.22E-11 | 0.028713 | 0.070837 | 0.013624 | 43 |
| rs11255790 | C | T | C | T | 0.007 | 0.041 | 4.71E-09 | 0.017918 | -0.01049 | 0.558132 | 34 |
| rs1157994 | G | A | G | A | 0.016 | 0.09 | 1.86E-08 | 0.034743 | 0.088348 | 0.010993 | 32 |
| rs11732089 | T | C | T | C | 0.008 | 0.115 | 7.43E-47 | 0.021634 | 0.012209 | 0.572516 | 207 |
| rs1203944 | C | T | C | T | 0.007 | 0.051 | 3.20E-13 | 0.017292 | -0.00918 | 0.595312 | 53 |
| rs12284404 | G | A | G | A | 0.007 | 0.067 | 1.05E-21 | 0.015224 | -0.02249 | 0.139666 | 92 |
| rs1265091 | T | C | T | C | 0.009 | 0.057 | 2.40E-10 | 0.020966 | -0.00024 | 0.990831 | 40 |
| rs12893151 | C | A | C | A | 0.008 | 0.062 | 9.19E-15 | 0.016074 | -0.01078 | 0.502614 | 60 |
| rs13015993 | A | G | A | G | 0.007 | 0.082 | 1.08E-31 | 0.014417 | -0.00056 | 0.969001 | 137 |
| rs13329353 | T | C | T | C | 0.007 | 0.061 | 2.93E-18 | 0.014781 | -0.01028 | 0.486818 | 76 |
| rs1663070 | C | T | C | T | 0.007 | 0.046 | 4.98E-11 | 0.0167 | -0.03934 | 0.018478 | 43 |
| rs17020122 | T | C | T | C | 0.011 | 0.104 | 3.24E-21 | 0.021942 | 0.006261 | 0.775368 | 89 |
| rs17477923 | T | C | T | C | 0.007 | 0.083 | 1.98E-32 | 0.015158 | -0.00237 | 0.875776 | 141 |
| rs17767491 | A | G | A | G | 0.007 | 0.088 | 3.03E-36 | 0.01528 | -0.0267 | 0.080588 | 158 |
| rs2127387 | A | G | A | G | 0.006 | 0.144 | 2.78E-127 | 0.014202 | 0.00018 | 0.989898 | 576 |
| rs2439301 | G | A | G | A | 0.008 | 0.059 | 1.64E-13 | 0.01577 | 0.004509 | 0.774952 | 54 |
| rs28502438 | T | C | T | C | 0.006 | 0.034 | 1.46E-08 | 0.014643 | -0.00495 | 0.73535 | 32 |
| rs30227 | C | T | C | T | 0.006 | 0.047 | 4.75E-15 | 0.0145 | 0.012851 | 0.375453 | 61 |
| rs334725 | A | G | A | G | 0.015 | 0.174 | 4.12E-31 | 0.049652 | -0.0373 | 0.452496 | 135 |
| rs398745 | C | A | C | A | 0.006 | 0.052 | 4.45E-18 | 0.014454 | 0.013976 | 0.333576 | 75 |
| rs4445669 | C | T | C | T | 0.006 | 0.04 | 2.62E-11 | 0.014042 | 0.009487 | 0.499306 | 44 |
| rs4804413 | T | C | T | C | 0.006 | 0.053 | 1.02E-18 | 0.014293 | -0.01736 | 0.224417 | 78 |
| rs4933466 | A | G | A | G | 0.006 | 0.04 | 2.62E-11 | 0.014254 | -0.01786 | 0.210293 | 44 |
| rs59381142 | G | A | G | A | 0.008 | 0.058 | 4.17E-13 | 0.017939 | -0.00281 | 0.875429 | 53 |
| rs7329958 | C | T | C | T | 0.007 | 0.044 | 3.26E-10 | 0.0147 | -0.00961 | 0.513349 | 40 |
| rs8015085 | A | G | A | G | 0.008 | 0.067 | 5.52E-17 | 0.018261 | 0.032522 | 0.074914 | 70 |
| rs9298749 | C | A | C | A | 0.006 | 0.039 | 8.03E-11 | 0.014135 | 0.009972 | 0.480493 | 42 |
| rs9381266 | T | C | T | C | 0.007 | 0.073 | 1.84E-25 | 0.017427 | 0.035435 | 0.042018 | 109 |
| rs9497965 | T | C | T | C | 0.006 | 0.044 | 2.24E-13 | 0.014894 | -0.02686 | 0.071359 | 54 |
| rs963384 | T | C | T | C | 0.006 | 0.035 | 5.43E-09 | 0.014047 | 0.010378 | 0.460048 | 34 |

SNP: single nucleotide polymorphism; EAE: effect allele exposure; OAE: other allele exposure; EAO: effect allele outcome; OAO: other allele outcome; SE: standard error;F:F value;


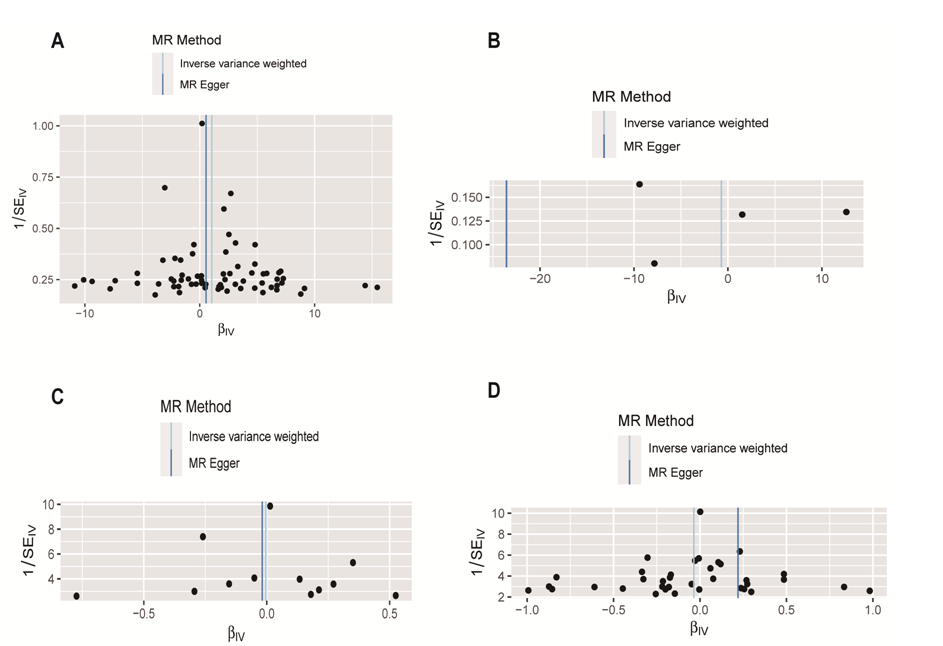


**Figure S1** **Funnle plots for Mendelian randomization (MR) analyses of the Causal relationship between thyroid dysfunction and hallux valgus.(A) Hypothyroidism-HV.(B)Hyperthyroidism-HV.(C)FT4-HV.(D)TSH-HV**


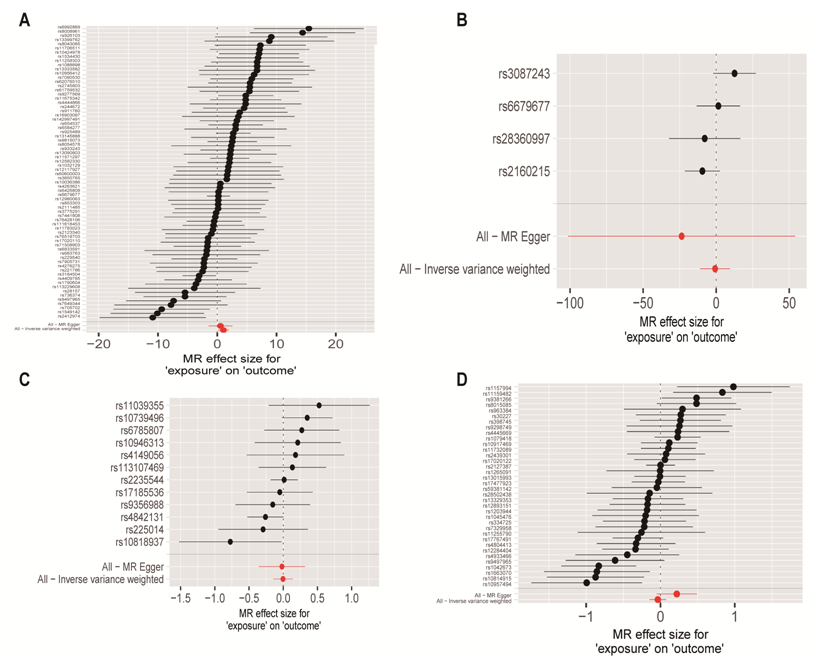


**Figure S2** **Forest plots for Mendelian randomization (MR) analyses of the Causal relationship between thyroid dysfunction and hallux valgus.(A) Hypothyroidism-HV.(B)Hyperthyroidism-HV.(C)FT4-HV.(D)TSH-HV**
